# Semi-experimental assessment of neutron equivalent dose and secondary cancer risk for off-field organs in glioma patients undergoing 18-MV radiotherapy

**DOI:** 10.1371/journal.pone.0271028

**Published:** 2022-07-29

**Authors:** Soheil Elmtalab, Iraj Abedi, Zahra Alirezaei, Mohammad Hossein Choopan Dastjerdi, Ghazale Geraily, Amir Hossein Karimi

**Affiliations:** 1 Department of Medical Physics, School of Medicine, Isfahan University of Medical Sciences, Isfahan, Iran; 2 School of Paramedicine, Bushehr University of Medical Sciences, Bushehr, Iran; 3 Reactor and Nuclear Safety Research School, Nuclear Science and Technology Research Institute, Isfahan, Iran; 4 Radiation Oncology Research Center, Cancer Institute, Tehran University of Medical Sciences, Tehran, Iran; 5 Department of Medical Physics and Biomedical Engineering, School of Medicine, Tehran University of Medical Sciences, Tehran, Iran; IPATIMUP/i3S, PORTUGAL

## Abstract

Neutron contamination as a source of out-of-field dose in radiotherapy is still of concern. High-energy treatment photons have the potential to overcome the binding energy of neutrons inside the nuclei. Fast neutrons emitting from the accelerator head can directly reach the patient’s bed. Considering that modern radiotherapy techniques can increase patient survival, concerns about unwanted doses and the lifetime risk of fatal cancer remain strong or even more prominent, especially in young adult patients. The current study addressed these concerns by quantifying the dose and risk of fatal cancer due to photo-neutrons for glioma patients undergoing 18-MV radiotherapy. In this study, an NRD model rem-meter detector was used to measure neutron ambient dose equivalent, H*(10), at the patient table. Then, the neutron equivalent dose received by each organ was estimated concerning the depth of each organ and by applying depth dose corrections to the measured H*(10). Finally, the effective dose and risk of secondary cancer were determined using NCRP 116 coefficients. Evidence revealed that among all organs, the breast (0.62 mSv/Gy) and gonads (0.58 mSv/Gy) are at risk of photoneutrons more than the other organs in such treatments. The neutron effective dose in the 18-MV conventional radiotherapy of the brain was 13.36 mSv. Among all organs, gonads (6.96 mSv), thyroid (1.86 mSv), and breasts (1.86 mSv) had more contribution to the effective dose, respectively. The total secondary cancer risk was estimated as 281.4 cases (per 1 million persons). The highest risk was related to the breast and gonads with 74.4 and, 34.8 cases per 1 million persons, respectively. Therefore, it is recommended that to prevent late complications (secondary cancer and genetic effects), these organs should be shielded from photoneutrons. This procedure not only improves the quality of the patient’s personal life but also the healthy childbearing in the community.

## 1. Introduction

Central nervous system (CNS) neoplasms account for 2.71% of cancer deaths. Among them, meningioma, originating from the meningeal layers around the brain, constitutes a large fraction (approximately 36.8%) of CNS neoplasms. Gliomas, initiating from glial cells, with an annual global prevalence rate of 6 per 1 million persons with a frequency of 75%, are considered the most common malignancies in the CNS [1]. Surgery as the main step of brain tumor treatment, followed by radiation therapy (RT) as an adjuvant therapy with significant progress can satisfactorily increase the average survival in people with malignancies [2].

Nowadays, the introduction of new techniques to radiotherapy has led to acceptable local tumor control and sparing of healthy tissues. In other words, factors such as conformity index (CI), homogeneity index (HI), and target coverage have been improved for such treatments [3–5]. Accordingly, modern RT techniques can increase RT patient survival by increasing treatment efficiency. Nevertheless, concerns about unwanted doses and the lifetime risk of fatal cancer remain critical for such patients [6, 7]. Recently, several studies have been designed for evaluating the out-of-field dose and lifetime risk of secondary cancer in radiotherapy [8–11]. Scientific evidence highlights the importance of these concerns.

Out-of-field doses in radiotherapy generally consist of scattered photons and neutron contamination at energies higher than 8 MV [12–14]. Briefly, scattered photons originate from three main sources, including the patient, leaking photons from the accelerator head, and the collimator [15, 16]. The linac head is the main source of neutron contamination. High-energy photons (> 8 MeV) have enough energy to overcome the binding energy of neutrons inside the nuclei [17]. Fast neutrons emitting from the accelerator head can directly reach the patient’s bed or slow down by interaction with the components of the treatment room, including walls, tables, and the like, and eventually, be converted into thermal neutrons [18]. Therefore, a spectrum of neutron energy is expected anywhere on the patient’s bed [19, 20].

Evidence suggests that the incidence of secondary malignancies may be associated with secondary radiation [21, 22]. Patients have a long life enough to experience secondary cancers by increasing their survival using modern radiotherapy techniques. Thus, determining the unwanted dose due to secondary radiation can help improve the quality of RT patients’ life, especially young adult patients. On the other hand, due to the inability of the treatment planning system (TPS) to calculate the dose of secondary radiation (scattered photons + neutron contamination) [6, 23, 24], it is impossible to determine the dose received by all critical organs of the body in routine clinic practices. This issue has attracted the attention of many researchers to reduce the out-of-field dose of the patient using experimental dosimetry methods or Monte Carlo codes. In addition, estimating the risk of secondary cancers can help physicians manage the prevalence of secondary malignancies.

The out-of-field dose and consequently secondary cancer risk received by the patient due to scattered photons in brain radiotherapy have been fully reported in the literature [7, 25, 26]. However, due to the average energy of neutrons emitting from the accelerator (1 MeV), the weight factor of radiation for such neutrons is approximately 20 times higher than that of X-rays or gamma photons [27]. Hence, it is expected to have more destructive biological effects. Considering that concerns about the neutron dose to the patient during this kind of treatment has increased, quantifying neutron doses can help raise public awareness in this regard.

Unfortunately, most of the neutron detectors used in dosimetry for the mixed fields (Photon + Neutron) are inevitably saturated by photons [28] which are challenging for the in-vivo dosimetry of neutron contamination in radiotherapy. Although neutron rem-meter detectors can separate neutrons from photons, they cannot be applied for in-vivo dosimetry due to their large size. The alternative method is the measurement of neutron ambient dose equivalent by these detectors and conversion of the measured dose to the clinical situation by dose conversion factors. Nevertheless, the confident usage of neutron rem-meter detectors for this purpose needs a validation process. In this regard, Elmtalab et al. [13] benchmarked the measured ambient dose equivalent by data calculated via Monte Carlo simulation. Additionally, Elmtalab et al. [13] reported the neutron equivalent dose only for a few superficial organs (lens and thyroid) without any secondary cancer risk assessment. Accordingly, a comprehensive report on neutron dose and secondary cancer risk should be provided for all out-of-field organs. The current study scientifically focused on this gap by quantifying the above-mentioned parameters for patients undergoing brain tumor radiotherapy using experimental dosimetry.

## 2. Material and methods

### 2.1 Ethics statement

The authors declare that this study does not involve human participants and only reports data obtained via in-vitro dosimetry. Therefore, participant consent was not requested in the research.

### 2.2 Treatment planning

**Fig 1:**
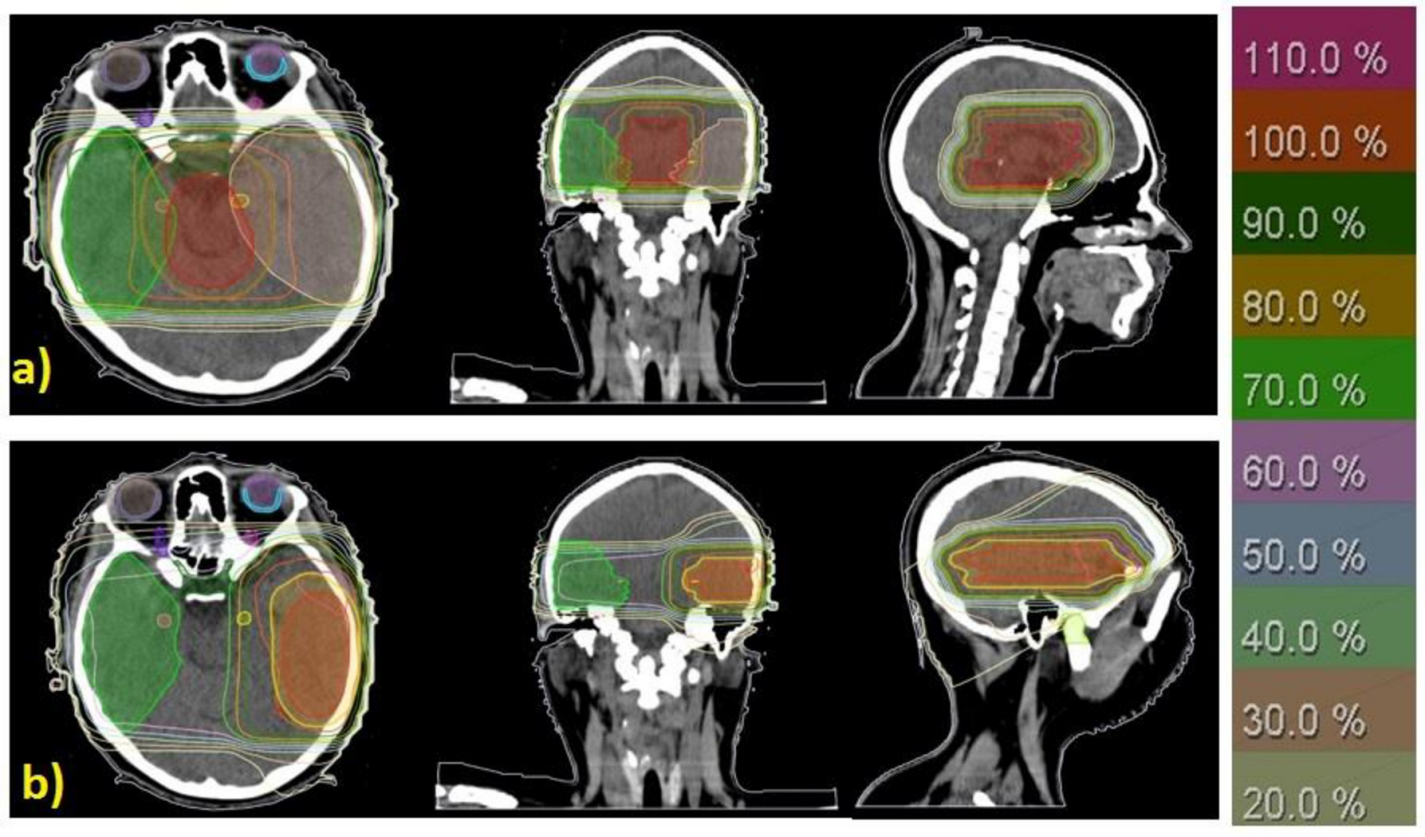
Dose distribution with a conformity index of 0.95 **(a)** for a deeply-seated tumor located in the midline via two lateral 18-MV beams and **(b)** for a superficial tumor located in the left temporal lobe via a lateral 6-MV beam (from the left) and an 18-MV beam (from the right). Planning Target Volume (PTV) = Clinical Target Volume (CTV) + 1 cm and CTV = Grass Target Volume + 0.6 cm.

Despite significant enhancements in treatment efficiency, new radiotherapy techniques are associated with more radiation exposure time for patients compared to conventional techniques [29]. In radiotherapy centers with high patient referral rates, conventional methods are inevitably preferred in treating some patients to save time. Due to the high penetration depth and skin-sparing properties, high-energy beams (>10 MV) can be effective in achieving the appropriate dose distribution in the conventional radiotherapy of brain tumors. Skin sparing is valuable in preserving patients’ hair, especially in women. High penetration depth is also important in the irradiation of deep tumors (usually gliomas). Even in the radiotherapy of superficial tumors (usual meningioma) located on one side of the brain (left or right), using a field with an 18-MV beam from the opposite side of the tumor is appropriate for creating a uniform dose distribution (Fig 1). In this study, treatment planning was performed for a large hypothetical tumor (~7 cm) located in the midline of a standard patient’s brain (70 kg) [30, 31] using TPS (PROWESS, version 5.5). The treatment plan consisted of two lateral fields with 18-MV beams that created a suitable dose distribution so that 95% of the planning target volume (PTV) received at least 95% of the prescribed dose. The dose to the organs at risk (OARs) was not more than the tolerated radiation dose (Table 1) [32]. The prescribed dose approved by an oncologist was 60 Gy (2 Gy/ per fraction).

**Table 1:** Tolerated dose of organs at risk in radiotherapy of head and neck area [32].

Organ	Dose limitation (Gy)
**Hippocampus**	6
**Optic nerves**	55
**Chiasm**	56
**Brainstem**	60
**Retina**	50

### 2.3 Detector and collaboration process

Ambient dose equivalent, H*(10), is defined by the International Commission on Radiation Units and Measurements (ICRU) as the equivalent dose that would be produced by the corresponding expanded and aligned field in the ICRU sphere at a depth of 10 mm on the radius opposing the direction of the aligned field [33].

**Fig 2:**
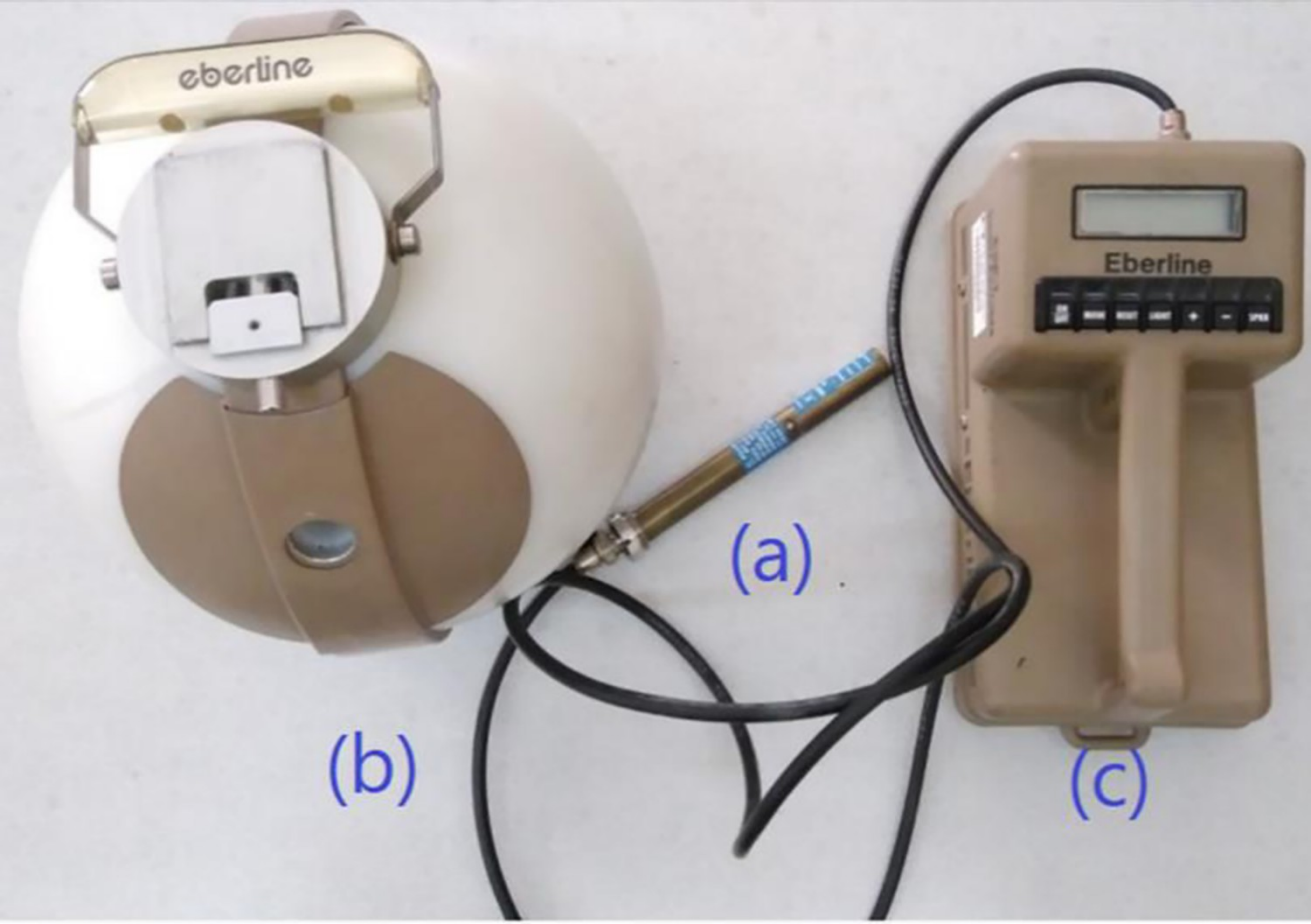
Neutron rem detector including **(a)** thermal neutron counter BF_3_, **(b)** polyethylene modulator and cadmium layer with suitable thickness, and **(c)** equipped with Eberline’s ASP-2e rate-meter with pulse height analysis capability (2000 V to create an appropriate differential pulse height distribution was adopted).

In this study, an NRD rem-meter detector (Thermo Electron Corporation, USA) was used to measure neutron equivalent dose. The detector includes a thermal neutron-sensitive proportional counter (BF_3_) centered on a standard 9-inch polyethylene moderator with a thin layer of cadmium (Fig 2). It can measure neutron equivalent dose in energies ranging from 0.025 eV (thermal) up to 10 MeV (epithermal and fast) with a sensitivity of 3000 counts/mrem independently of the energy and direction of radiation (within 10%). In addition, the detector is equipped with an Eberline’s ASP-2e electronic system that can calculate H*(10) using the flux-to-dose conversion coefficients provided by the International Commission on Radiological Protection’s system, as well as the gamma rejection capability with 500 R/h, which is suitable for use in neutron fields that are saturated with photons [34].

In our previous study [13], detector calibration in a neutron field (nearly equal to the irradiation field) generated by a miniature neutron source reactor (MNSR) was described in detail. In summary, the MNSR is a 30-kW tank-in-pool research reactor. The calibration was performed using an external neutron beam with the flux of 2.7 E+5 n/ (cm^2^.s) in the outlet (at full power of the reactor) [35].

### 2.4 Depth and horizontal distance corrections

Due to its large size, the detector is incapable of measuring neutron equivalent dose directly inside an anthropomorphic phantom. Therefore, the measurements were performed at different horizontal distances along the central axis of the beam. Then, distance and depth corrections were applied by knowing the horizontal distance of the target organ from the central axis of the beam and its depth from the surface (Table 2) [36]. This correction is applied because the neutron dose decreases with distance from the central axis of the photon beam and increases with the depth in the tissue.

**Table 2:** Position of the center of the organs: Horizontal distance of the organs from the central axis of the beam and the depth of the organs from the phantom surface. This information is extracted from Howell et al’s study [36] using an Eclipse measuring instrument for an Alderson Radiation Therapy Phantom Female. In this table, the organs are classified into three levels of depth (superficial, middle, and deep).

Organ	Distance (cm)	Depth (cm)
^**a**^ Brain***	5	13.0
**Salivary glands** **	8.38	6.0
**Thyroid** *	15.28	2.0
**Esophagus** ***	29.88	13.5
Breast*	29.88	2.0
**Lung** ***	30.88	12.5
**Cord** ***	32.28	16.0
**Heart** **	34.88	9.5
**Stomach** ***	43.88	10.5
**Spleen** **	43.88	9.0
**Liver** **	44.38	8.0
**Pancreas** ***	45.88	11.0
**Kidney** ***	48.88	12.5
**Colon** **	57.88	9.5
**Bladder** **	78.18	8.5
**Gonads** *	79.88	1.0
**Rectum** ***	79.38	14.0
**Femoral head** ***	83.88	11.5

^a^ Healthy brain tissue, whose horizontal distance from the central axis of the beam, was considered after the edge of the field.

* Surface depth organs (0 cm ≤ x ≤ 5 cm).

** Medium depth organs (5 cm < x ≤ 10 cm).

***Deep organs (10 cm < x ≤ 15 cm).

Neutron spectrum out-of-field does not change significantly (less than 10%) in bin distance [20–40] and [40–60] cm from the central axis of the beam [36]. Accordingly, it is assumed that the measured H*(10) at 20 and 60 cm far away from the isocenter are appropriate for estimating the dose received by organs in the distance bins of [5–40] and [40–80] cm, respectively. To decrease statistical uncertainties, measurements were repeated three times at each point.

The neutron equivalent dose received by each organ can be estimated concerning the depth of each organ from the surface (Table 2), the average neutron energy at the patient bed (about 0.5 MeV) [13, 28, 37], and by applying depth dose corrections to the measured H*(10) provided by d’Errico et al (Table 3) [38].

**Table 3:** Neutron absorbed dose reported by d’Errico et al. As a function of the initial neutron energy (0.5 MeV) and the depth in a phantom 30 × 30 × 20 cm^3^ [38].

Depth (cm)	D_n_ (pGy/cm^2^)
**1**	18.39
**2**	16.11
**3**	12.50
**4**	9.31
**5**	7.16
**6**	5.75
**8**	2.79
**10**	1.40
**12**	0.69
**14**	0.43
**16**	0.15

### 2.5 Irradiation set-up

**Fig 3:**
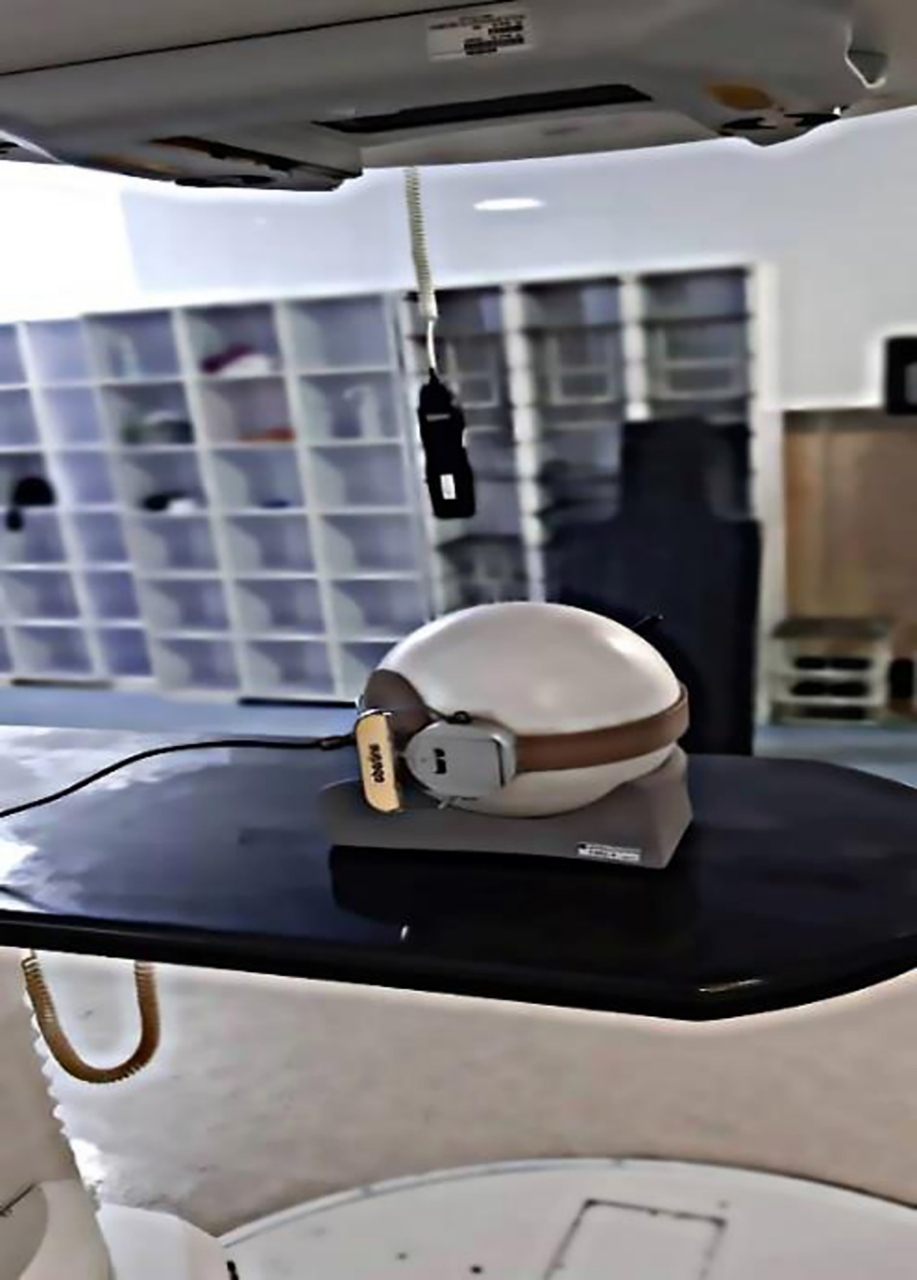
Detector set-up for measurement of neutron ambient dose equivalent at the isocenter under a Source to Surface Distance (SSD) of 95 cm. A similar procedure was employed also for measurements at the 20 and 60 cm far away from the isocenter.

Irradiation (2 Gy) in a static field of 10 × 10 cm^2^ was performed at 0° gantry angle by an 18- MV Siemens Oncor linear accelerator located at a bunker with dimensions 12 × 14 × 5.5 m^3^ and walls made of ordinary concretes (with a density of 2.35 g/cm^3^) in Milad Hospital, Isfahan (Fig 3).

The neutron spectrum is independent of the radiation angle (Gantry angle) and the direction of the dosimeter (within 10%). The symmetric geometry of the treatment room keeps the neutron spectra at the patient table nearly independent of the gantry angle. Accordingly, to simplify the calculations, instead of using two lateral fields with equally weighted monitor units (Fig 1), only one treatment field was employed for the irradiation of the detector.

### 2.6 Effective dose and risk of secondary cancer

By determining the neutron equivalent dose (*H_T_*) received by each organ and applying the tissue weighting factors (*W_T_*) proposed by NCRP 116 [39], the effective dose (E) was calculated as follows:

$$E=\sum W_{T}\times H_{T}$$

NCRP 116 assigns a W_T_ to the bladder, bone marrow, bone surface, breast, esophagus, colon, liver, lung, gonads, skin, stomach, and thyroid. Its value for other remainder organs is reported to be 0.05. According to NCRP 116, organs such as the adrenals, brain, small intestine, large intestine, kidney, muscle, pancreas, spleen, thymus, and uterus are defined as remainder organs. However, not all these organs can be identified in the phantom. Therefore, among the contoured organs in Table 2, the organs with undetermined W_T_ factors were considered as the remainder organ. It should be noted that determining the neutron equivalent dose is more complex for bone marrow, bone surface, and skin. For this purpose, Howell et al. [36] suggested a suitable method through which the neutron equivalent dose received by organs such as the brain, breast, heart, spinal cord, and the femoral head is used to estimate the neutron equivalent dose of the head, upper limb-girdle, sternum/ribs, vertebrae, and sacrum/ lower girdle, respectively. The estimated doses are then weighted based on the active red bone marrow distribution of a 40-year-old male [30]. Accordingly, the percentage of the active bone marrow in the head, upper limb-girdle, sternum, ribs, vertebrae, sacrum, and lower girdle are 13.1, 6.2, 3.4, 14.1, 10.9, 13.9, and 26.1%, respectively. Bone surface neutron equivalent dose was estimated using the average equivalent dose of upper limb-girdle, sternum, ribs, vertebrae, sacrum, and lower girdle. Given that the skin is a superficial organ (extends up to a depth of 4 mm), the neutron equivalent dose of the skin was measured via average H*(10) at distances of 20 and 60 cm far from the isocenter. Considering that the area of the skin inside the treatment field is extremely small, we ignored the intra-field area in dose calculations.

Finally, the risk of secondary cancer in critical out-of-field organs was assessed by employing the secondary cancer risk coefficients proposed by NCRP 116 to the obtained neutron equivalent doses and considering the assumptions in determining the neutron equivalent dose of remainder organs, bone marrow, bone surface, and skin.

### 2.7 Photoneutrons vs. scattered photons

As mentioned earlier, scattered photons are a part of secondary radiation that causes unwanted doses to the healthy organs/tissues outside the treatment field. Recently, in a similar treatment plan (18-MV radiotherapy of the brain area under two lateral fields), out-of-field dose and consequently the risk of secondary cancer due to scattered photons in the Medical Internal Radiation Dose Phantom have been calculated using Monte-Carlo simulation [26]. To provide a practical radiation protection viewpoint, the results of this study were qualitatively compared with those of the present study in terms of dose distribution in the body and high-risk organs of secondary cancers.

## 3. Results

The measured H*(10) at the patient table in 20 cm (corresponding to the organs close to the treatment field 5 cm ≤ x ≤ 40 cm) and 60 cm (corresponding to the more distant organs) far away from the isocenter is reported in Table 4. The results were also compared with those obtained in a similar condition by Zanini et al [40]. The H*(10) at the isocenter found in our previous measurement [13] was also reported to make the comparisons more meaningful.

**Table 4:** Neutron ambient dose equivalent, H*(10), in different distances at the patient table under a 10 × 10 cm^2^ treatment field when 1 Gy photon dose was delivered to the isocenter. The results also were compared with Zanini et al’s study [40].

H*(10) (mSv/Gy)							
Study	LINAC	Dosimeter	Distance from isocenter (cm)				
			0	15	20	50	60
**This study**	Siemens Oncor (18-MV)	NRD model neutron rem-meter	1.30 ± 0.14	-	0.71 ± 0.12	-	0.58 ± 0.10
**Zanini et al.**	Elekta (18-MV)	Bubble Detector	1.7	0.9	-	0.4	-

Table 5 provides comprehensive information on the neutron equivalent dose received by different organs during high-energy conventional brain tumor radiation therapy.

**Table 5:** Neutron equivalent dose (H_T_), effective dose, and risk of secondary cancer & genetic effects for a glioma patient undergoing 18-MV radiotherapy when 60-Gy photon dose is delivered to the brain with two lateral 10 × 10 cm^2^ treatment fields. Tissue weighting factor (W_T_) and risk coefficients were employed based on NCRP 116 recommendation [39].

Organ		H_T_ (mSv)	W_T_	Risk coefficients (10^−2^ Sv^-1^)	Secondary cancer risk (per 1 million persons)
**Bladder**		4.20 ± 0.42	0.05	0.30	12.60 ± 1.30
**Bone marrow**		6.00 ± 0.80	0.12	0.50	30.00 ±4.00
**Bone surface**		9.00 ± 1.20	0.01	0.05	4.50 ±0.60
**Breast** ^**a**^		37.20 ± 1.16	0.05	0.20	74.40 ± 2.32
**Esophagus**		1.20 ± 0.15	0.05	0.30	3.60 ± 0.45
**Colon**		3.00 ± 0.30	0.12	0.85	25.50 ±2.60
**Liver**		5.40 ± 0.54	0.05	0.15	8.10 ±0.81
**Lung**		1.20 ± 0.15	0.12	0.85	10.20 ±1.28
**Gonads**		34.80 ± 3.50	0.20	0.10	34.80 ±3.50
**Skin** ^**b**^		38.70 ± 0.22	0.01	0.02	7.74± 0.04
**Stomach**		2.40 ± 0.24	0.12	1.10	26.40 ± 2.70
**Thyroid**		37.20 ± 4.00	0.05	0.08	29.76 ±3.20
**Remainder**	**Rectum**	0.60 ± 0.10	0.05	0.05	13.80 ± 0.18
	**Femoral head** ^**a**^	1.20 ± 0.20			
	**Salivary Glands**	13.20 ± 1.70			
	**Spleen**	3.60 ± 0.40			
	**Heart** ^**a**^	4.20 ± 0.54			
	**Pancreas**	2.40 ± 0.30			
	**Kidney**	0.60 ± 0.10			
	**Brain** ^**a**^	1.20 ± 0.15			
	**Cord** ^**a**^	0.60 ± 0.08			
**Effective dose (mSv)**		13.36 ± 1.29			
**Total secondary cancer risk (per 1 million persons)**		281.40 ± 23.00			
**Genetic effects (per 1 million persons)**		384.00 ±35.00			

^**a**^ These organs were used to predict the dose received by the Head, Upper limb-girdle, Sternum/Ribs, Vertebrae, and Sacrum/Lower girdle and finally to estimate the dose reached to the bone marrow (taking into account the weight distribution of bone marrow in a forty-year male body) and Bone surface.

^**b**^ The dose received by the skin is considered as the average ambient dose equivalent at intervals of 20 cm and 60 cm far from the isocenter.

^**c**^ Genetic effects (per million persons) were calculated using the neutron equivalent dose of gonads (34.80 ± 0.00 mSv) and the genetic effects coefficient (1.00 10^−2^ Sv^-1^) extracted from NCRP 116 [39].

**Fig 4:**
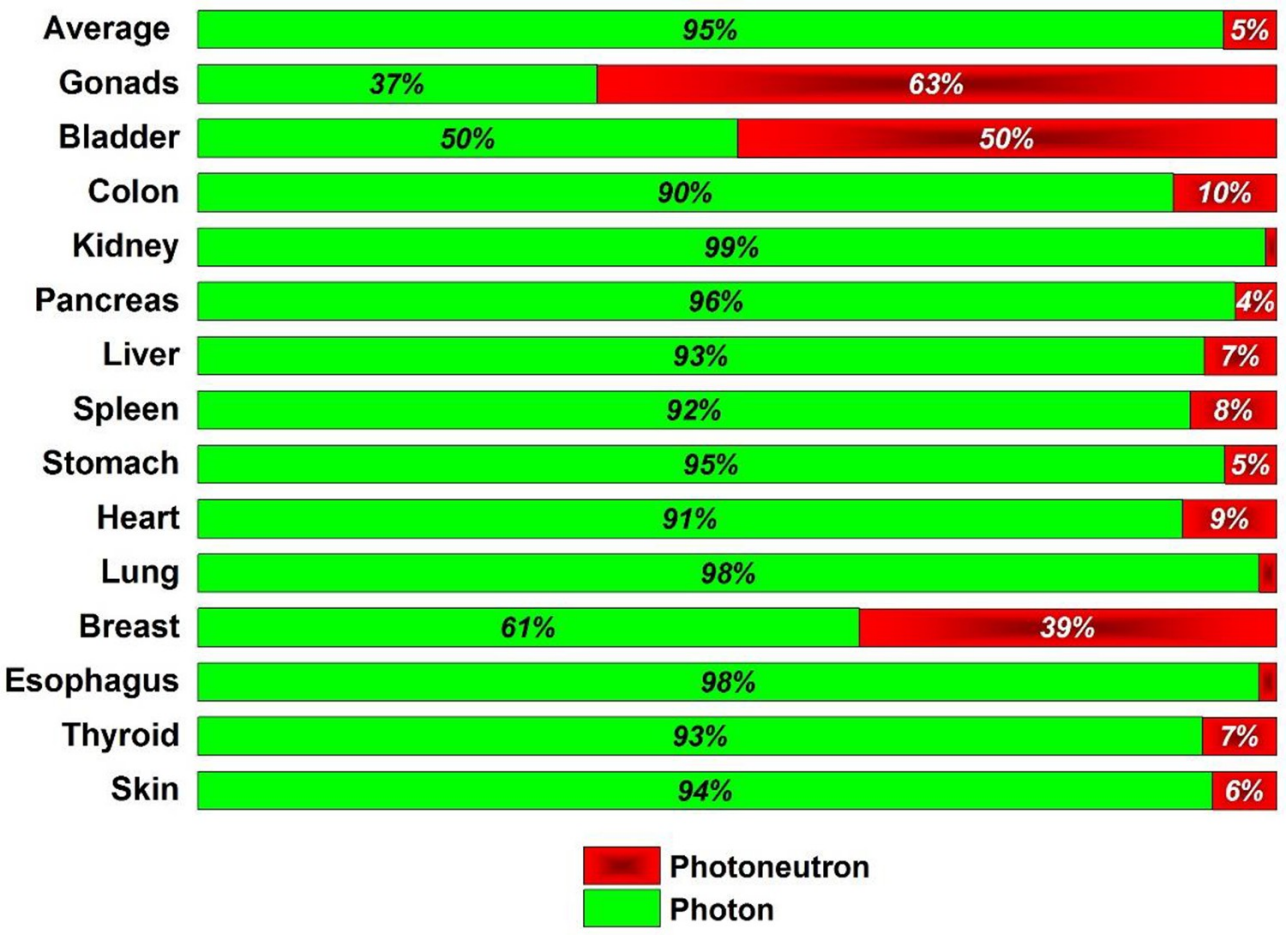
Contribution of photoneutrons and scattered photons to the total equivalent dose received by several out-of-field organs in glioma patients undergoing 18-MV radiotherapy.

Fig 4 illustrates a qualitative comparison between the out-of-field dose of scattered photons and photoneutrons as a function of the horizontal distance of the organ from the isocenter.

## 4. Discussion

Based on data in Table 4, the H*(10) rapidly decreases from 1.3 to 0.71 mSv/Gy by increasing the distance from the isocenter. Then, an extremely smoother reduction from 0.71 to 0.58 mSv/Gy can be observed in H*(10). Accordingly, the assumption that the fluctuation of H*(10) at 20-cm intervals outside the treatment field is less than 10% seems reasonable. Table 4 also presents that the measured values for H*(10) are in good agreement with those reported by Zanini et al. Nevertheless, in the study of Zanini et al., H*(10) at the isocenter is nearly 23% more than the value of 1.3 mSv/Gy measured using the NRD rem-meter in this study; this difference is because the bubble detector is potentially saturated by photons. Additionally, evidence represents that Siemens linear accelerators generate less neutron contamination than Elekta machines [41]. It was also reported that the measurement method can cause a difference of 20% in neutron dosimetry [28, 36]. Nonetheless, the mean H*(10) outside the treatment field is almost the same between the two studies (a difference of less than 0.01 mSv/Gy).

According to Table 5, the average neutron equivalent dose intake by the body was 0.16 mSv/Gy, ranging from 0.01 to 0.64 mSv/Gy. The skin (0.64 mSv/Gy), thyroid (0.62 mSv/Gy), breast (0.62 mSv/Gy), and gonads (0.58 mSv/Gy) received the highest neutron equivalent dose, respectively. Given that H*(10) decreases with horizontal distance from the isocenter (Table 4), at the first glance, it is expected that the neutron equivalent dose for organs far from the isocenter to be less pronounced than those which are close to the treatment field. Nevertheless, the neutron equivalent dose received by the gonads, at a distance of 80 cm far from the isocenter, is about 30 times more than the neutron equivalent dose received by the brain (Table 5). The reason is that the neutron equivalent dose decreases due to increasing the horizontal distance from the isocenter and attenuation through passing the body. The attenuation of neutrons with depth is a more predominant factor compared to horizontal distance from the isocenter, thus superficial organs such as the gonads (depth 1 cm) and breasts receive more neutron equivalent dose in comparison to the brain as a deep organ (the depth of its geometric center is 13 cm above the body surface). Accordingly, it seems useful to classify the neutron equivalent dose received by organs into superficial, middle, and deep areas. The average neutron equivalent dose for the surface, middle, and deep organs is 0.61, 0.09, and 0.02 mSv/Gy, respectively. A comparison of these values shows the significant dependence of neutron equivalent dose on the depth.

The obtained data (Table 5) further indicate that the neutron effective dose in the high-energy conventional radiotherapy of the brain is 0.23 mSv/Gy. Gonads (52%), thyroid (14%), breasts (14%), and bone marrow (5%) have more contribution to the effective dose compared to other organs, respectively. As previously mentioned, in addition to the neutron equivalent dose of organs, the W_T_ of each organ plays a key role in calculating the effective dose. Therefore, although the skin receives a similar neutron equivalent dose compared to the thyroid or breast (0.64 ~ 0.62, both in mSv/Gy), its contribution to the effective dose is less pronounced (~ 5 times) because of its lower W_T_ (0.05 > 0.01).

The ultimate goal of this study was to assess the risk of secondary cancer. Based on the NCRP 116 report [39], the risk of secondary cancer is determined by the dose received by each organ and the intrinsic susceptibility of the organ (secondary cancer risk coefficients). Considering the two above-mentioned factors, the risk of secondary cancer due to photoneutrons was evaluated for different organs of a typical glioma patient receiving a 60 Gy treatment dose via 18-MV photons. The total secondary cancer risk was estimated to be approximately 281.4 cases (per 1 million persons). The highest risk was related to the breast (74.4 cases per 1 million persons), gonads (34.8 cases per 1 million persons), bone marrow (30 cases per 1 million persons), and thyroid (29.76 cases per 1 million persons), respectively. Recently, it has been found that for glioma patients, scattered photons lead to the highest risk of secondary cancer of the thyroid, lung, and stomach, respectively [26]. In fact, concerning the scattered photons, the thyroid (as a vital organ close to the treatment field) is significantly at risk of secondary cancer. However, as an internal organ, it cannot be shielded from the scattered photons. On the other hand, in terms of photoneutron contamination, gonads and breasts can be protected with radiation protection techniques such as shielding or patient positioning. For a glioma patient, putting the patient’s hands on the chest can reduce the neutron equivalent dose reached to the breast. The radiation protection of the gonads looks more significant since the genetic effects on the gonads (384 cases per 1 million persons) are remarkable. Regarding the severe attenuation of neutrons with depth, a gonad shield (bolus or paraffin plates) with an appropriate thickness is suggested to be located on the body surface in the region of the gonads.

In contrast to scattered photons, the reduction of neutron equivalent dose by increasing horizontal distance from the isocenter is extremely slow (Fig 4). In other words, a sharp reduction in the photon dose can be expected by increasing the distance from the isocenter compared to the neutron equivalent dose. Furthermore, the contribution of scattered photons to the unwanted dose to the organs at risk is more remarkable than that of photoneutrons. Nevertheless, the diagram shows that the dose ratio (neutron to photon) increases with distance from the edge of the treatment field until finally, the neutron equivalent dose prevails.

Neutron dosimetry is usually associated with at least 10% uncertainty, thus the estimation of secondary cancer risk strongly depends on accurate information about dose values [28]. In addition, neutron dose values rely on treatment plan details. The variety of models used for risk assessment can also lead to differences in estimations. Therefore, an accurate estimation of secondary cancer risk is still a challenge. Nevertheless, in this study, by determining the neutron equivalent dose for the off-field organs, the authors attempted to provide reliable information about the levels of secondary cancer risk for a typical glioma patient undergoing 18-MV radiotherapy. This information can help physicians employ radiation protection considerations for improving the quality of life of patients with brain tumors after treatment.

## 5. Conclusion

This study sought to estimate the risk of secondary malignancies due to the neutron contamination of the linear accelerator head during the conventional treatment of brain tumors with 18-MV photons. After applying depth corrections to the measured H*(10), the neutron equivalent dose received by the OARs was assessed, followed by estimating the risk of secondary cancer for OARs by applying malignancy risk coefficients based on the NCRP 116 protocol. Additionally, the effective dose received due to neutrons during such treatment was also calculated using tissue weighting factors proposed by the mentioned protocol.

Among all organs, skin, thyroid, breast, and gonads had the highest potential of receiving the neutron equivalent dose, respectively. Moreover, organs such as gonads, thyroid, breast, and bone marrow had the highest contribution to the effective dose, respectively. Breast, bone marrow, gonads, and thyroid were potentially at high risk of secondary cancer induced by photoneutrons. Therefore, gonads, thyroid, skin, breast, and bone marrow are the critical organs that photoneutrons can contaminate in brain radiation therapy. However, it does not mean that radiation protection considerations must be performed on all these organs to reduce the neutron equivalent dose; because among them, the skin and thyroid receive a large fraction of unwanted dose from the scattered photons (an inevitable internal source for unwanted dose). The breast and gonads are the only organs that receive a predominant dose due to photoneutrons. Therefore, it is recommended that these organs should be shielded from photoneutrons during the high-energy radiotherapy of brain tumors to prevent late complications (secondary cancer and genetic effects). This procedure not only improves the quality of the patient’s personal life but also increases healthy childbearing in the community.

## Supporting information

S1 File Data set corresponds to Table 5. (XLSX)

S2 File Photoneutrons vs. scattered photons: Unwanted dose received by several out-of-field organs in glioma patients undergoing 18-MV radiotherapy. This file corresponds to Fig 4 in the paper. Photon data were extracted from Reference [26] in the paper.
(XLSX)

## References

[1] Reynoso-Noverón N, Mohar-Betancourt A, Ortiz-Rafael J. Epidemiology of brain tumors. Principles of Neuro-Oncology: Springer; 2021. p. 15–25.

[2] Juratli TA, Schackert G, Krex D. Current status of local therapy in malignant gliomas—a clinical review of three selected approaches. Pharmacology & therapeutics. 2013;139(3):341–58. 23694764 10.1016/j.pharmthera.2013.05.003

[3] Lu S-H, Cheng JC-H, Kuo S-H, Lee JJ-S, Chen L-H, Wu J-K, et al. Volumetric modulated arc therapy for nasopharyngeal carcinoma: a dosimetric comparison with TomoTherapy and step-and-shoot IMRT. Radiotherapy and Oncology. 2012;104(3):324–30. doi: 10.1016/j.radonc.2011.11.017 22236614

[4] Siddhesh Tryambake M, Vikram R, Dhavale A, Mali S. Clinical Outcomes and Dosimetric Analysis of 3D Conformal, Intensity-Modulated and Volumetric Arc Radiation Therapy in Post-operative Oral cavity Cancers–A Single Institution Retrospective Audit. 2021.

[5] Weidlich GA, Hacker F, Bellezza D, Maguire P, Gardner EA. Ventricular tachycardia: a treatment comparison study of the cyberknife with conventional linear accelerators. Cureus. 2018;10(10). doi: 10.7759/cureus.3445 30555760 PMC6294279

[6] Colnot J, Zefkili S, Gschwind R, Huet C. Out‐of‐field doses from radiotherapy using photon beams: A comparative study for a pediatric renal treatment. Journal of Applied Clinical Medical Physics. 2021;22(3):94–106. doi: 10.1002/acm2.13182 33547766 PMC7984471

[7] Elmtalab S, Abedi I. Investigating the out-of-field doses and estimating the risk of secondary thyroid cancer in high-grade gliomas radiation therapy with modulated intensity and 3D-conformal: a phantom study. International Journal of Radiation Research. 2021;19(3):569–74.

[8] Geraily G, Elmtalab S, Mohammadi N, Alirezaei Z, Martinez-Ovalle S, Jabbari I, et al. Monte Carlo evaluation of out-of-field dose in 18 MV pelvic radiotherapy using a simplified female MIRD phantom. Biomedical Physics & Engineering Express. 2021;8(1):015004. doi: 10.1088/2057-1976/ac35a1 34727526

[9] Karimi AH, Chegeni N, Jabbari I, Hassanvand M. The effect of neutron contamination on probability of secondary cancer in radiotherapy of pelvic region with 18-MV photons. Journal of Isfahan Medical School. 2019;37(519):222–7.

[10] Karimi AH, Mirian SF, Mahmoudi F, Geraily G, Vega-Carrillo HR, Mohiuddin M. Feasibility of 18-MV grid therapy from radiation protection aspects: unwanted dose and fatal cancer risk caused by photoneutrons and scattered photons. Computer methods and programs in biomedicine. 2022;213:106524. doi: 10.1016/j.cmpb.2021.106524 34818621

[11] Wen L, Zhong G, Ren M. Increased risk of secondary bladder cancer after radiation therapy for endometrial cancer. Scientific Reports. 2022;12(1):1–10. 35058550 10.1038/s41598-022-05126-w PMC8776857

[12] Banaee N, Goodarzi K, Nedaie HA. Neutron contamination in radiotherapy processes: a review study. Journal of Radiation Research. 2021;62(6):947–54. doi: 10.1093/jrr/rrab076 34467374

[13] Elmtalab S, Shanei A, Choopan Dastjerdi MH, Brkić H, Abedi I, Amouheidari A. Determination of the Neutron Contamination During Brain Radiotherapy Using a Moderated-Boron Trifluoride Detector and the MCNP Monte Carlo Code. Radiation Protection Dosimetry. 2022;198(3):129–38. doi: 10.1093/rpd/ncac001 35137234

[14] Sánchez‐Nieto B, Medina‐Ascanio K, Rodríguez‐Mongua J, Doerner E, Espinoza I. Study of out‐of‐field dose in photon radiotherapy: A commercial treatment planning system versus measurements and Monte Carlo simulations. Medical physics. 2020;47(9):4616–25. doi: 10.1002/mp.14356 32583441 PMC7586840

[15] López‐Guadalupe VM, Rodríguez‐Laguna A, Poitevin‐Chacón MA, López‐Pineda E, Brandan ME. Out‐of‐field mean photon energy and dose from 6 MV and 6 MV FFF beams measured with TLD‐300 and TLD‐100 dosimeters. Medical Physics. 2021;48(11):6567–77. doi: 10.1002/mp.15233 34528262

[16] Schneider CW, Newhauser WD, Wilson LJ, Kapsch R-P. A physics-based analytical model of absorbed dose from primary, leakage, and scattered photons from megavoltage radiotherapy with MLCs. Physics in Medicine & Biology. 2019;64(18):185017. 31535622 10.1088/1361-6560/ab303a

[17] Farhood B, Ghorbani M, Goushbolagh NA, Najafi M, Geraily G. Different methods of measuring neutron dose/fluence generated during radiation therapy with megavoltage beams. Health physics. 2020;118(1):65–74. doi: 10.1097/HP.0000000000001130 31764421

[18] 60 MSC. Neutron Contamination from Medical Electron Accelerators: Recommendations of the National Council on Radiation Protection and Measurements: National Council on Radiation; 1984.

[19] Razghandi S, Karimi-Shahri K, Firoozabadi M. Evaluation of neutron spectra and dose equivalent from a Varian 2100C/D medical linear accelerator: Monte Carlo simulation and a literature review. Radioprotection. 2021;56(2):93–101.

[20] Sohrabi M, Hakimi A. Photoneutron spectrometry by novel multi-directional spherical neutron spectrometry system. Scientific Reports. 2021;11(1):1–17. 33547354 10.1038/s41598-021-81529-5 PMC7864933

[21] Jin MC, Qian ZJ, Megwalu UC. Risk of second primary malignancies after external beam radiotherapy for thyroid cancer. Anticancer Research. 2022;42(3):1359–65. doi: 10.21873/anticanres.15605 35220228

[22] Sitathanee C, Tangboonduangjit P, Dhanachai M, Suntiwong S, Yongvithisatid P, Rutchantuk S, et al. Secondary cancer risk from modern external-beam radiotherapy of prostate cancer patients: Impact of fractionation and dose distribution. Journal of Radiation Research. 2021;62(4):707–17. doi: 10.1093/jrr/rrab038 33993271 PMC8273793

[23] Howell RM, Scarboro SB, Kry S, Yaldo DZ. Accuracy of out-of-field dose calculations by a commercial treatment planning system. Physics in Medicine & Biology. 2010;55(23):6999. doi: 10.1088/0031-9155/55/23/S03 21076191 PMC3152254

[24] Kavousi N, Nedaie HA, Gholami S, Esfahani M, Geraily G. Evaluation of dose calculation algorithms accuracy for eclipse, PCRT3D, and monaco treatment planning systems using IAEA TPS commissioning tests in a Heterogeneous Phantom. Iranian Journal of Medical Physics. 2019;16(4):285–93.

[25] De Saint-Hubert M, Verellen D, Poels K, Crijns W, Magliona F, Depuydt T, et al. Out-of-field doses from pediatric craniospinal irradiations using 3D-CRT, IMRT, helical tomotherapy and electron-based therapy. Physics in Medicine & Biology. 2017;62(13):5293. doi: 10.1088/1361-6560/aa6c9e 28398210

[26] Elmtalab S, Karimi AH, Mehr FSK, Zamani H, Abedi I, Pashaei F. Estimating Radiotherapy-Induced Secondary Cancer Risk Arising from Brain Irradiation at High Energy: A Monte Carlo Study. Frontiers in Biomedical Technologies. 2022;9(1).

[27] Nath R. Neutron measurements around high energy x-ray radiotherapy machines: a report of Task Group 27, Radiation Therapy Committee, American Association of Physicists in Medicine: American Inst. of Physics; 1987.

[28] Karimi AH, Brkić H, Shahbazi-Gahrouei D, Haghighi SB, Jabbari I. Essential considerations for accurate evaluation of photoneutron contamination in Radiotherapy. Applied Radiation and Isotopes. 2019;145:24–31. doi: 10.1016/j.apradiso.2018.12.007 30572262

[29] Kinhikar RA, Pawar AB, Mahantshetty U, Murthy V, Dheshpande DD, Shrivastava SK. Rapid Arc, helical tomotherapy, sliding window intensity modulated radiotherapy and three dimensional conformal radiation for localized prostate cancer: A dosimetric comparison. Journal of cancer research and therapeutics. 2014;10(3):575. doi: 10.4103/0973-1482.138200 25313742

[30] Ellis R. The distribution of active bone marrow in the adult. Physics in Medicine & Biology. 1961;5(3):255. 10.1088/0031-9155/5/3/302 13726497

[31] Valentin J. Basic anatomical and physiological data for use in radiological protection: reference values: ICRP Publication 89. Annals of the ICRP. 2002;32(3–4):1–277. 14506981

[32] Cox J. Toxicity criteria of the Radiation Therapy Oncology Group (RTOG) and the European Organization for Research and Treatment of Cancer (EORTC) 92–04. Int J Radiat Oncol Biol Phys. 1995;31:134–6. 10.1016/0360-3016(95)00060-C 7713792

[33] Protection ICoR. Conversion coefficients for use in radiological protection against external radiation: International Commission on Radiological Protection; 1996.

[34] Hankins D. MODIFIED-SPHERE NEUTRON DETECTOR. Los Alamos National Lab.(LANL), Los Alamos, NM (United States), 1966.

[35] Dastjerdi MC, Mokhtari J, Asgari A, Ghahremani E. A neutron radiography beamline relying on the Isfahan Miniature Neutron Source Reactor. Nuclear Instruments and Methods in Physics Research Section A: Accelerators, Spectrometers, Detectors and Associated Equipment. 2019;928:20–5.

[36] Howell RM, Hertel NE, Wang Z, Hutchinson J, Fullerton GD. Calculation of effective dose from measurements of secondary neutron spectra and scattered photon dose from dynamic MLC IMRT for, and beam energies. Medical physics. 2006;33(2):360–8. doi: 10.1118/1.2140119 16532941

[37] Brkić H, Ivković A, Kasabašić M, Sovilj MP, Jurković S, Štimac D, et al. The influence of field size and off-axis distance on photoneutron spectra of the 18 MV Siemens Oncor linear accelerator beam. Radiation measurements. 2016;93:28–34.

[38] d’Errico F, Luszik-Bhadra M, Nath R, Siebert B, Wolf U. Depth dose-equivalent and effective energies of photoneutrons generated by 6–18 MV X-ray beams for radiotherapy. Health physics. 2001;80(1):4–11. doi: 10.1097/00004032-200101000-00003 11204115

[39] NCRP. Limitation of exposure to ionizing radiation: NCRP Report No. 116; 1993.

[40] Zanini A, Durisi E, Fasolo F, Visca L, Ongaro C, Nastasi U, et al. Neutron spectra in a tissue equivalent phantom during photon radiotherapy treatment by LINACS. Radiation protection dosimetry. 2004;110(1–4):157–60. doi: 10.1093/rpd/nch205 15353639

[41] Howell RM, Kry SF, Burgett E, Hertel NE, Followill DS. Secondary neutron spectra from modern Varian, Siemens, and Elekta linacs with multileaf collimators. Medical physics. 2009;36(9Part1):4027–38. 10.1118/1.3159300 PMC2738742 19810475

